# Gene‐expression contributions to specific and shared regional vulnerabilities to amyloid, tau, and neurodegeneration in Alzheimer’s disease

**DOI:** 10.1002/alz70862_110034

**Published:** 2025-12-23

**Authors:** Cecilia Boccalini, Ines Hristovska, Débora E. Peretti, Augusto J. Mendes, Max Scheffler, Giovanni B Frisoni, Valentina Garibotto

**Affiliations:** ^1^ University of Geneva, Geneva, Switzerland Switzerland; ^2^ Clinical Memory Research Unit, Department of Clinical Sciences, Lund University, Lund Sweden; ^3^ Laboratory of Neuroimaging and Innovative Molecular Tracers (NIMTlab), Geneva University Neurocenter and Faculty of Medicine, University of Geneva, Geneva Switzerland; ^4^ Laboratory of Neuroimaging of Aging (LANVIE), University of Geneva, Geneva Switzerland; ^5^ Division of Radiology, Geneva University Hospitals, Geneva Switzerland; ^6^ Geneva Memory Center, Department of Rehabilitation and Geriatrics, Geneva University Hospitals, Geneva Switzerland; ^7^ Faculty of Medicine, University of Geneva, Geneva, Geneva Switzerland; ^8^ Division of Nuclear Medicine and Molecular Imaging, Geneva University Hospitals, Geneva Switzerland

## Abstract

**Background:**

Protein deposition and neurodegeneration differently affect the brain spatially and temporally in Alzheimer’s disease (AD). Here we used imaging transcriptomics to understand the biological and molecular properties underlying regional variability of neuroimaging phenotypes of amyloid, tau, and neurodegeneration assessed by PET and MRI.

**Method:**

Brain patterns were estimated by contrasting imaging data between AD patients and healthy controls from two independent cohorts for replication (Geneva Memory Clinic and ADNI). Regional gene expression profiles were derived from brain‐wide microarray measurements provided by the Allen human brain atlas (AHBA). Hypothesis‐driven analyses assessed the spatial association between neuroimaging patterns and gene expression (gene‐to‐biomarker associations) for selected candidate genes for AD. Over‐representation analysis (ORA) and gene set enrichment analysis (GSEA) were used to characterize molecular properties and biological pathways of genome‐wide gene sets associated with regional AD pathologies in a data‐driven manner.

**Result:**

Regional patterns showed the highest amyloid load in frontal, parietal, and lateral temporal lobes, whereas tau deposition was most pronounced in medial temporal lobes and lateral temporoparietal areas. Neurodegeneration patterns were instead less widespread, involving mainly temporoparietal areas. Specific patterns of amyloid, tau and neurodegeneration were differently associated with AD‐related genes. ORA and GSEA revealed that genes implicated in different aspects of protein synthesis (e.g. cytosolic ribosome, mitochondrion organization, and RNA metabolic processes) as well as immune regulation and neuroinflammation correlated exclusively with amyloid load, whereas genes involved in the synaptic organization, transmission, and function were associated to the severity of amyloid, tau, and neurodegeneration pathologies. GSEA confirmed that the gene‐to‐tau and gene‐to‐atrophy associations were related to similar biological pathways involving synaptic signaling and organization, while gene‐to‐hypometabolism associations were more related to cellular processes.

**Conclusion:**

Selective AD vulnerabilities were differently related to specific gene expression and molecular‐biological properties, with a large set of genes associated with amyloid accumulation and a subset of genes conferring additional vulnerability to downstream tau. Our findings suggest that the spatial and temporal decoupling between amyloid deposition, tau deposition and neurodegeneration is explained by differential genetic expression but that shared mechanisms link upstream amyloid with subsequent tau pathology and loss of neuronal integrity.

